# Labrador sea water spreading and the Atlantic meridional overturning circulation

**DOI:** 10.1098/rsta.2022.0189

**Published:** 2023-12-11

**Authors:** Isabela Alexander-Astiz Le Bras

**Affiliations:** Department of Physical Oceanography, Woods Hole Oceanographic Institution, Woods Hole, MA, USA

**Keywords:** deep ocean circulation, high-latitude oceanography, water mass analysis, ocean dynamics

## Abstract

In 1982, Talley and McCartney used the low potential vorticity signature of Labrador Sea Water (LSW) to make the first North Atlantic maps of its properties. Forty years later, our understanding of LSW variability, spreading time scales and importance has deepened. In this review and synthesis article, I showcase recent observational advances in our understanding of how LSW spreads from its formation regions into the Deep Western Boundary Current and southward into the subtropical North Atlantic. I reconcile the fact that decadal variability in LSW formation is reflected in the Deep Western Boundary Current with the fact that LSW formation does not control subpolar overturning strength and discuss hypothesized connections between LSW spreading and decadal Atlantic Meridional Overturning Circulation variability. Ultimately, LSW spreading is of fundamental interest because it is a significant pathway for dissolved gasses such as oxygen and carbon dioxide into the deep ocean. We should hence prioritize adding dissolved gas measurements to standard hydrographic and circulation observations, particularly at targeted western boundary locations.

This article is part of a discussion meeting issue ‘Atlantic overturning: new observations and challenges’.

## 1. Introduction

Labrador Sea Water (LSW) is formed by dramatic wintertime convection and spreads southward in the deep limb of the Atlantic Meridional Overturning Circulation (AMOC). The southward spreading of this oxygen- and carbon-rich water mass is thought to prevent large-scale hypoxia in the North Atlantic and store anthropogenic carbon away from the atmosphere for hundreds of years [1,2]. Thus, understanding how LSW spreads into the deep North Atlantic and how this might change in the future is a critical component of understanding our changing climate.

The fact that LSW spreads into the deep Atlantic was suggested by Wüst [3], who observed a core of high oxygen waters at about 2000 m depth focused at the western boundary. After World War II, Stommel and Arons developed a theory for a Deep Western Boundary Current (DWBC) along which waters such as LSW could spread [4]. The existence of this current was confirmed by the coordinated hydrographic and float measurements of Swallow & Worthington [5].

**Figure 1.**
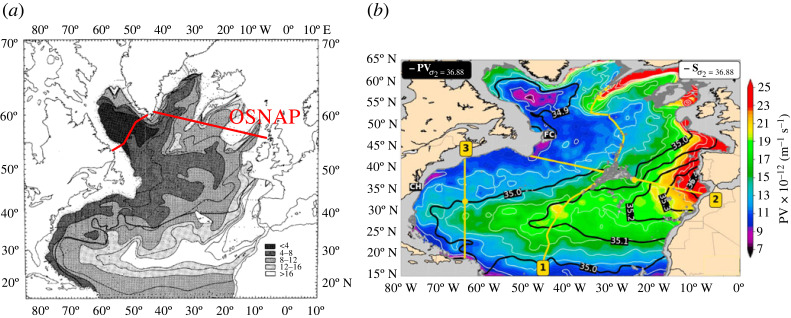
Potential vorticity of LSW from Talley & McCartney [6] (*a*) and altered from Biló & Johns [7] (*b*). Both figures show that LSW is focused on the western edge of the basin, and has interior pathways east of Flemish Cap (FC) and north of Cape Hatteras (CH). The figures use the same units (shown in the colourbar). Talley and McCartney used the PV minimum itself to define LSW, while Biló and Johns show PV on the $\sigma_{2}=36.88\;\mathrm{kg}\;m^{-3}$ surface. Biló and Johns also show salinity contours on this surface, highlighting that low PV LSW waters are significantly fresher than the Mediterranean Overflow Water in the same density range. The approximate location of the OSNAP line is shown in red in the left panel. Both panels used with permission; (*a*) American Meteorological Society, (*b*) John Wiley & Sons, Inc.

In 1982, Talley & McCartney made detailed maps of LSW in the North Atlantic based on its characteristic low potential vorticity (PV) [6] (figure 1*a*). LSW has low PV because it is formed through deep convection, which creates a thick, unstratified layer [8,9]. LSW is also anomalously fresh and cold as winter atmospheric temperatures mix fresh Arctic waters to great depths. Talley and McCartney’s maps show that LSW spreads primarily, but not exclusively, along the western boundary of the North Atlantic and found that the LSW PV, temperature and salinity minimum increased as LSW spread southward. They hypothesized that temporal changes in LSW formation would be reflected in the spatial patterns of LSW properties, but also noted that mixing must be important because downstream density variations were twice those of density variations in the source regions.

More recently, Biló & Johns constructed updated maps of LSW in the North Atlantic based on Argo float data [7] (figure 1*b*). The overall spreading patterns are similar to those in Talley and McCartney’s maps, but with much more data (above 2000 m), the updated maps are smoother, and Biló and Johns were able to quantify the transport of interior spreading pathways east and west of the Mid-Atlantic Ridge. These important interior pathways were hypothesized to be linked to the Gulf Stream’s recirculation gyres by Lozier [10], and have been studied in detail, particularly from a Lagrangian perspective [10–17].

Though the DWBC is not its only spreading pathway, LSW is focused on the western boundary of the North Atlantic, and the DWBC is the fastest route for LSW into the subtropics [12]. Furthermore, several modelling and reanalysis-based studies have suggested that LSW density anomalies propagating southward in the DWBC can affect AMOC strength through thermal wind adjustment [18,19]. Because the strength of LSW formation and hence its density is controlled by the strength of the North Atlantic Oscillation (NAO) on decadal time scales [20], this would project onto decadal AMOC variability.

In order for this mechanism to hold, however, there are two minimum requirements. The first is that LSW is consistently exported from its formation regions, i.e. that variability in LSW formation makes it out of the Labrador Sea. The second is that spreading in the DWBC is coherent enough that LSW property anomalies are preserved as they move southward. Both of these steps continue to be debated in the scientific literature, and both are fundamental to our understanding of LSW spreading, regardless of the links with AMOC variability.

In this paper, I present the current state of observational evidence for the southward spreading of LSW anomalies in the DWBC structured around these two requirements. First, I review recent advances in our understanding of how LSW is exported into the DWBC from its formation regions (§2). In this section, I also reconcile the fact that decadal variability in LSW formation is reflected in the Labrador Sea’s DWBC with the fact that LSW formation does not impact subpolar overturning strength. I then synthesize the literature on LSW spreading into the subtropical North Atlantic and suggest that water mass anomaly tracking, anthropogenic tracers and Lagrangian studies generally agree on LSW transit times in the DWBC (§3). Finally, I discuss the connections between LSW spreading and the AMOC, which remain uncertain (§4), and end with a final synthesis and outlook (§5).

## 2. Labrador Sea Water export from formation regions

**Figure 2.**
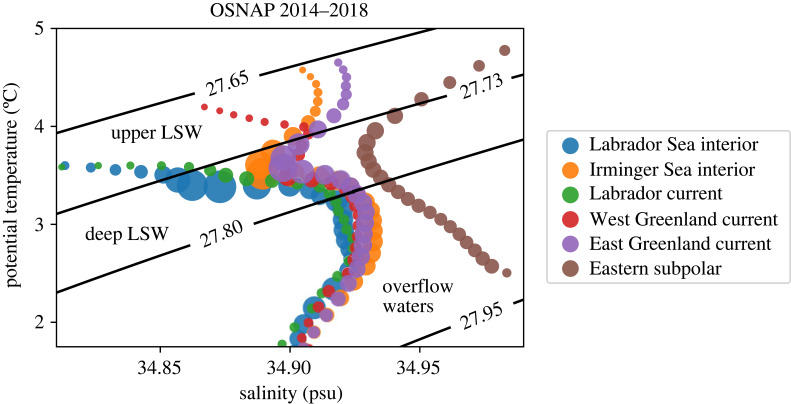
Time-mean temperature salinity diagram of waters in the lower limb of the overturning circulation across the OSNAP array (2014–2018). The size of the circle corresponds to the amount of water in each density class. Water mass boundary potential density anomalies are labelled. Locations are indicated in figure 3; eastern subpolar refers to everything east of the mid-Atlantic Ridge.

LSW forms from the Labrador Sea to the Irminger Sea [21–24]. LSW formed by convection in the basin interiors is sometimes referred to as ‘deep’ LSW (dLSW) to distinguish it from ‘upper’ LSW (uLSW), which forms within or near the western boundary current of the Labrador Sea [25]. Some papers argue that uLSW can be formed in the central Labrador Sea under weak forcing [26–29], while others classify LSW formed in different years using a vintage system [23]. The Overturning in the Subpolar North Atlantic Program (OSNAP) moored observations, which have offered the first across-subpolar basin year-round view of water mass properties and transports [30,31], confirm that dLSW is the most abundant temperature-salinity class in the subpolar gyre (figure 2) [32]. Though convection in one basin may precondition the other, the densest, coldest and freshest dLSW is found in the interior of the Labrador Sea (figure 2) [23]. It has been hypothesized that a water mass analogous to uLSW forms in the western Irminger Sea [33]; however, these waters still bear the warm and salty signature of subtropical Atlantic Waters, unlike the uLSW in the Labrador Sea (figure 2). The properties within the dLSW density class are much more similar across the OSNAP array, as these waters are only in contact with the atmosphere for brief periods during the winter and are sheltered from the atmosphere as they move between basins [16].

One of the major findings of the OSNAP is that the strength and variability of subpolar overturning is dominated by overturning east of Greenland, with minimal contribution from the Labrador Sea [34]. The overturning strength is generally reported as the transport at the density of maximum overturning for each portion of the OSNAP array at each time step. Using this metric, the time-mean overturning from 2014 to 2018 across the full OSNAP array is very similar to that across OSNAP East (16.6 and 16.8 Sv, respectively) [30]. If, instead, the mean isopycnal of maximum overturning is used ($\sigma_{\theta }=27.65\;\mathrm{kg}\;m^{-3}$), the time-mean overturning across the full OSNAP array is 14.8 Sv. Furthermore, the overturning streamfunction is generally accumulated from the surface downward and the 1.6 Sv northward flow through OSNAP East and 1.6 Sv southward flow through the Labrador Sea are apparent at the bottom of the streamfunctions.

**Figure 3.**
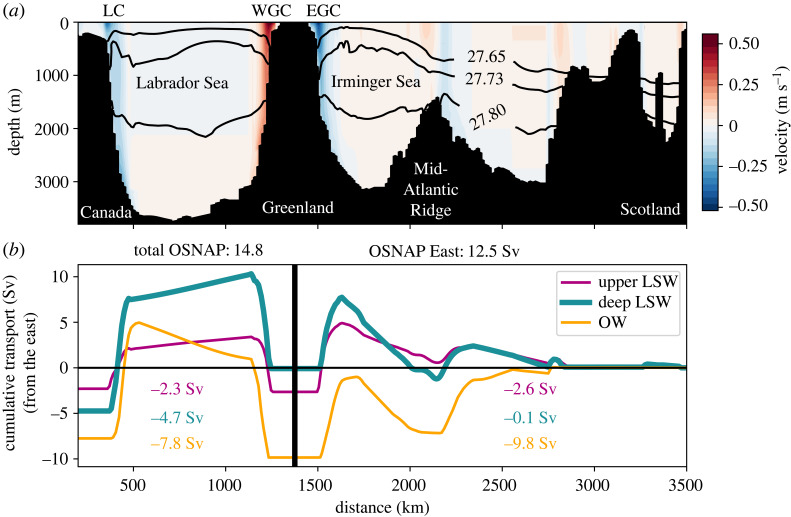
(*a*) Time-mean (2014–2018) velocity across the OSNAP array, with potential density surfaces indicating the time-mean isopycnal of maximum overturning and the upper and lower boundaries of deep LSW labelled in figure 2. Geographical features and currents are labelled; LC, Labrador Current; WGC, West Greenland Current; EGC, East Greenland Current. (*b*) Transport accumulated from east to west for the water masses in the subpolar overturning circulation’s lower limb, i.e. between the isopycnals shown in (*a*). Water mass cumulative transports for both OSNAP East and the full array are reported within the figure colour-coded as in the legend and each total is in the title.

Here, we simply examine the flow below the mean isopycnal of maximum overturning, which is equivalent to accumulating the streamfunction from the seafloor upward, and the contribution of the Labrador Sea becomes more apparent. The lower limb defined in this way gains over 2 Sv in the Labrador Sea; the lower limb transport is 12.5 Sv across OSNAP East and 14.8 Sv across the full array (figure 3). Furthermore, the water masses in this lower limb converge into the dLSW water mass class in the Labrador Sea. There is only 0.1 Sv of southward transport of dLSW across OSNAP East, while there is 4.7 Sv across the full OSNAP array (figure 3). These additional 4.6 Sv are associated with 2.3 Sv of new transformation across the isopycnal of maximum overturning to the dLSW density range and 0.3 Sv of transformation from the uLSW to the dLSW density range, as well as 2 Sv of dense-to-light (likely mixing driven) transformation from OW to dLSW. This convergence into the dLSW density class has been noted in previous studies. Though their focus is on the strong density-compensation associated with converting warm, salty waters to cold, fresh waters in the Labrador Sea, Zou *et al.* [35] show (in their fig. 1b) that there is about 4 Sv of convergence into the LSW density range [35]. Evans *et al.* [36] further highlight the role of mixing in driving this water mass convergence [36].

The OSNAP observations show that LSW is exported in the boundary currents of the Irminger and Labrador Seas (figure 3). However, in order for decadal variability in LSW formation to be reflected downstream, it must be exported in a relatively direct manner. If LSW experiences significant mixing or a wide range of pathway lengths into boundary currents, it is conceivable that decadal signatures could be mixed away before LSW is exported from the subpolar North Atlantic. However decade-long observations in the Labrador Sea suggest that decadal signatures are in fact exported in the DWBC. For example, Palter *et al.* [37] examined quasi-Lagrangian float observations in the Labrador Sea’s boundary current and found the thickest layers of LSW exiting the DWBC in the late 1990s when the thickest layers of LSW were formed by intense deep convection in the central Labrador Sea [37]. Into the early 2000s, as LSW formation slowed, the thickness of LSW exiting the DWBC decreased. They note, however, that many of the thick layers of LSW in the DWBC appear to be ventilated within the boundary current. Yashayaev & Loder [23] similarly found that decadal temperature variability in LSW formation regions (at 1500 m) is mirrored at the western boundary (at 1000 m) [23]. Of course, given that there is some direct ventilation of the boundary current, this could also result from the boundary being exposed to the same decadal atmospheric forcing.

Recent OSNAP observations have allowed a closer look at LSW export into boundary currents. Le Bras *et al.* [33] showed that some waters formed by deep convection in the interior of the Irminger Sea are mixed into the boundary current within months of their formation [33]. Le Bras *et al.* refer to the water mass formed by convection in the Irminger Sea as Irminger Sea Intermediate Water (ISIW) to emphasize its formation history. ISIW is slightly lighter than LSW, but has substantial overlap with its density class and is similarly split into ‘upper’ and ‘deep’ classes that are formed near the boundary and in the interior, respectively. The western Irminger Sea moorings they analyse recorded during a transitional period from little convection prior to 2014 to strong convection starting in early 2015. At the beginning of the moored record, there are no anomalously thick layers of ISIW in the interior or in the boundary current of the Irminger Sea. Once convection starts, thick, fresh and cold deep ISIW is found both within the interior and in the western boundary current (their figs. 2 and 3). The deep ISIW in the interior is thicker, fresher and colder than that found in the boundary current, indicating that there is some stirring or mixing as waters move from the interior into the boundary current. Deep ISIW does not appear to be ventilated within the boundary current, unlike upper ISIW, whose formation is thought to be impacted by slantwise convection in the boundary current [38].

**Figure 4.**
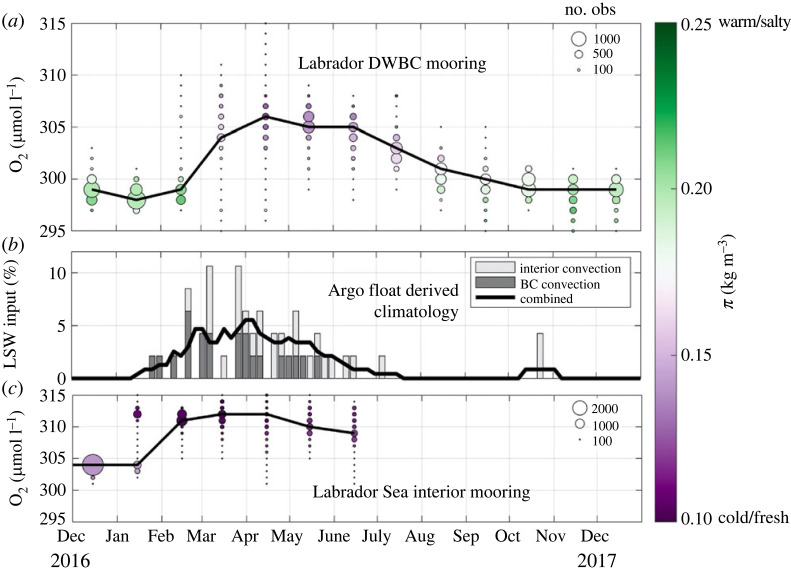
(*a*) Monthly distribution of oxygen at about 600 m depth in the core of the DWBC in the western Labrador Sea (K9 mooring on the 2900 m isobath). The circle size indicates the number of observations in each oxygen bin and the black line highlights the oxygen bin with the most measurements each month. Colours correspond to the mean spiciness in each oxygen bin. (*b*) Seasonal cycle of climatological LSW input to the boundary current estimated using Argo float profiles and trajectories from 2000 to 2020. Light grey bars indicate floats that recorded LSW formation inshore of the 3000 m isobath and dark grey bars indicate floats that measured interior convection before entering the boundary current. Grey bars are shown in 5 day bins and the black line is the total LSW input smoothed with a 25 day running average. (*c*) As in (*a*) for oxygen measured at 500 m in the deep convection area of the Labrador Sea by the SeaCycler mooring. (Adapted from Koelling *et al.* [39]).

Koelling *et al.* [39] investigated LSW export in the DWBC using the first year-round oxygen observations in the western Labrador Sea [39]. They found that the oxygen in the DWBC increases from February into April and is accompanied by a cooling and freshening, which is characteristic of LSW (figure 4*a*). Oxygen increases in the LSW formation region several months earlier, from December into February (figure 4*c*). Koelling *et al.* connect the boundary and the interior using an Argo float analysis in which they track floats that measure LSW in the boundary current and identify when the LSW was last in contact with the atmosphere. They find that LSW is formed within the boundary current just west of the interior convection site starting in late January, as suggested by earlier studies [40,41]. LSW formed in the interior enters the boundary current from late February into July (figure 4*b*), which is consistent with the oxygen observations [42].

The Koelling *et al.* [39] analysis found that about half the floats containing LSW in the boundary current measured formation onshore of the 3000 m isobath, and about half measured formation in the interior (figure 4*b*). However, they caution against over-interpreting these statistics as this analysis is based on 47 Argo floats over 20 years. Models can help build a more quantitative picture of LSW export from the Labrador Sea. Brandt *et al.* [43], using a 1 year run of an eddy resolving model, found that there is about equal transport of upper and deep LSW in the Labrador Current, and that about half of the waters in both density classes were ventilated within 1 year [43]. More recently, MacGilchrist *et al.* [44] and Georgiou *et al.* [45] used Lagrangian model analyses to identify where deep waters are subducted in the Labrador Sea [44,45]. While Georgiou *et al.* again found that about half of the LSW exiting the Labrador Sea are subducted in the boundary current and half in the interior, MacGilchrist *et al.* found that 70% of the subduction occurs within the boundary current and only 30% in the interior, indicating that these results are model dependent and require further investigation as well as careful comparison with observations.

The model studies of Brandt *et al.* and Georgiou *et al.* generally agree with the Koelling *et al.* and Le Bras *et al.* observations on the timing of export of LSW types: LSW formed near the boundary is exported within months of formation, while export of LSW formed in the interior begins later and lasts into the summer months. Georgiou *et al.* [45] further suggest that density variations in the DWBC are primarily controlled by waters that are exchanged with the interior and that the location of exchange with the boundary is important. They find that waters that enter the boundary current from the interior near western Greenland take about 2.5 years longer to exit the Labrador Sea than those entering the boundary current on the Canadian side. This would act to further decrease the magnitude of the decadal variability in the LSW properties that are exported in the DWBC.

Observations and models are starting to converge on a consistent picture of how LSW is exported from the subpolar North Atlantic in the DWBC. LSW formed by deep convection in the interior of the Labrador and Irminger Seas is thought to be stirred into the boundary current along isopycnals [33,39,46–49]. A lighter variant of LSW forms directly in the boundary current and is exported within a few months of formation [33,39]. Though only a small fraction of the deep LSW formed in the interior is exported in the boundary current and most of the upper LSW formed in boundary currents is exported directly, most studies indicate that these two variants are exported in roughly equal proportions [39,43,45]. Deep LSW tends to have a more characteristic temperature, salinity and density signature and is thought to be the primary control on density variation in the DWBC [45]. Decadal variability of LSW in the boundary current tends to mirror decadal variability in LSW formation [23,37,50]. Though a generally consistent picture is emerging, observations remain sparse and models disagree on the role of boundary convection as well as the effect of stirring and mixing encountered by LSW along its export pathways [44,45]. In 2020, moored oxygen sensors were deployed on OSNAP moorings across the Labrador Sea and western Irminger Sea through GOHSNAP (Gasses in the Overturning and Horizontal Subpolar North Atlantic Program) [51]. These new measurements will elucidate LSW export in boundary currents across the subpolar North Atlantic in the coming years in conjunction with ongoing modelling studies.

The DWBC is not the only known spreading pathway for LSW. It is well known that a significant amount of LSW spreads eastward into the subpolar North Atlantic [16,52,53], as well as southward via interior pathways [13]. However here we focus on boundary currents as they are the only potentially coherent pathway into the subtropics. Other southward interior pathways, or recirculations into the eastern subpolar, are subject to more stirring and mixing. In the following section, we focus on how decadal variability in LSW formation is reflected in the continued southward spreading of LSW in the DWBC.

## 3. Labrador Sea Water spreading in the Deep Western Boundary Current

The DWBC is a bottom-intensified current that is most effectively monitored using moored arrays. As such, measurements of the DWBC are particularly sparse. The longest running DWBC observations are at the exit of the Labrador Sea at 53N and began in 1997 [42]. Moving southward, there have been shorter term arrays at the Flemish Cap (47N), and the Grand Banks (42N) [54,55]. In the subtropical North Atlantic, the DWBC has been measured by the Line W moorings from 2004 to 2014 (39N) [56] and by the RAPID array since 2004 (26N) [57]. There are also measurements in the South Atlantic [58,59], as well as east of Greenland [60], but these are excluded from the present discussion as the focus is on LSW. Overall, the North Atlantic moored arrays measure a DWBC with peak velocities of about $20\text{--}40\;\mathrm{cm}\;s^{-1}$ and a transport of about 20–30 Sv. The reader is referred to Toole *et al.* [56] for a detailed comparison and discussion of local recirculation patterns [56].

**Figure 5.**
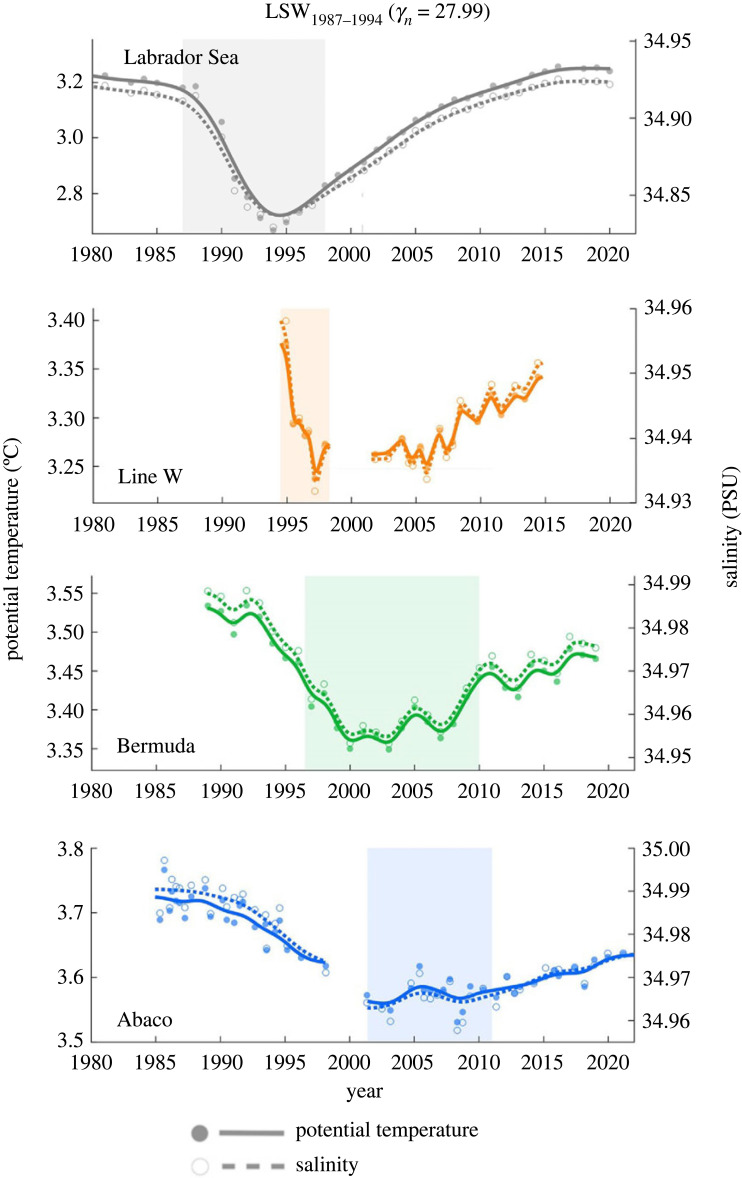
Water mass properties from shipboard hydrographic measurements throughout the North Atlantic on the $\gamma_{n}=27.99$ neutral density surface, which is thought to represent the dense LSW formed from 1987 to 1994. Potential temperature and salinity are represented as indicated in the legend, with values indicated on the left y-axis and right y-axis, respectively. Note the difference in axis range between locations. The shaded region highlights the passage of the temperature and salinity minimum in each panel based on visual assessment. (Adapted from Chomiak *et al.* [61]).

LSW spreading rates can be estimated by tracking water mass property anomalies along the path of the DWBC. Chomiak *et al.* [61] recently confirmed a spreading rate of about $2.5\;\mathrm{cm}\;s^{-1}$ [50,61–64]. As in previous studies, Chomiak *et al.* follow the cold, fresh anomaly associated with intense deep convection in the Labrador Sea in the early-mid 1990s. They track this anomaly along a neutral density level to Line W (39N), where it arrives about 5 years later and to Abaco (26N), where it arrives about 10 years later (figure 5). As previously discussed by Le Bras *et al.* [50], the gap in Line W observations complicates interpretation of the arrival time of the intense cold, fresh LSW anomaly to this latitude. As the anomaly travels southward, its amplitude decreases and it becomes saltier due to stirring with Mediterranean waters and other vintages of LSW (figure 5). Interestingly, in order to align the anomaly patterns, Chomiak *et al.* apply a neutral density offset of $-0.015\;\mathrm{kg}\;m^{-3}$ to the datasets downstream of the Labrador Sea. This suggests that there is strong mixing at some point between the Labrador Sea convection site and Line W that does not persist between Line W and Abaco. Entrainment into the DWBC in the Labrador Sea is a potential candidate, as well as increased mixing in the Tail of the Grand Banks region [13,65], but this requires further investigation. Chomiak *et al.* also suggest slower spreading in a lighter LSW water mass class, potentially due to increased stirring in shallower waters.

LSW spreading rates can also be estimated using anthropogenic tracers such as Chlorofluorocarbons (CFCs), Tritium, Helium and Iodine. Analyses of anthropogenic tracer observations suggest about a $1\;\mathrm{cm}\;s^{-1}$ DWBC spreading rate from the Labrador Sea to the subtropical North Atlantic [65–68], less than half of what has been found using water mass property anomalies. Because spreading in the DWBC is in an intermediate mixing regime characterized by transit time distributions with a long tail, this apparent disagreement can be resolved by considering the differences in initial conditions. As explained by Waugh & Hall [69], transit times estimated from tracers with an initial condition that resembles exponential growth, such as many anthropogenic tracers, will tend to be biased slow if not analysed carefully. This is because longer transit times will only ever lower the tracer concentration observed downstream. On the other hand, for a tracer with approximately periodic initial conditions, such as water mass salinity, longer transit times will act to shift the phase of the signal and decrease its amplitude. When LSW DWBC anthropogenic tracer and water mass property observations at Line W are analysed using a consistent framework that accounts for their initial conditions, there is good agreement between the calculated advection and mixing time scale estimates [50,70].

Comparisons with results from Lagrangian studies are complicated by the difference in perspective. While Lagrangian methods can be used to estimate transit time distributions directly, Eulerian observations can only reveal how a tracer was affected by a transit time distribution. At the same time, Lagrangian float observations are not sufficient to calculate robust spreading statistics and must be paired with complementary model analyses to estimate transit time distributions [17,71]. Nevertheless, Gary *et al.* [72] were able to reconcile tracer and float observations of LSW spreading in the DWBC using a detailed modelling analysis [72]. In sum, there is general agreement in the observational literature on LSW spreading rates in the DWBC though the results may appear different at first glance. Anomaly spreading rates are generally about $2.5\;\mathrm{cm}\;s^{-1}$ and waters are subject to significant stirring so that only significant hydrographic anomalies are detectable downstream.

## 4. Links to the Atlantic Meridional Overturning Circulation

Jackson *et al.* [18] suggest that decadal AMOC variability is linked to variability in LSW formation through thermal wind adjustment [18]. They propose that as positive LSW density anomalies propagate southward along the western boundary they are associated with positive AMOC anomalies. They associate the AMOC decline observed at RAPID with the weakening of LSW formation, connected with a lag of about 10 years. This spreading rate is consistent with the observations discussed in §3. Analyses of the Line W moorings found a decrease in LSW thickness during a similar time period, as well as a decline in DWBC strength [50,56]. To address whether these changes were associated with an AMOC decline at this latitude, Le Bras *et al.* [73] combined Line W mooring data with satellite altimeter and Argo float data to construct an AMOC time series at 35N [73]. They found that the $0.7\;\mathrm{Sv}\;{\mathrm{yr}}^{-1}$ slowing of the DWBC from 2004 to 2014 is associated with a statistically significant $0.3\;\mathrm{Sv}\;{\mathrm{yr}}^{-1}$ slowing of the AMOC. They find this AMOC decline at 35N in the ECCO state estimate as well.

Desbruyères *et al.* [74] suggest that decadal AMOC variability is set by water mass transformation at high latitudes more generally rather than LSW formation specifically, building on Walin and Marsh [74–76]. In order for this relationship to hold, they assume that the accumulation of waters within density classes in the subpolar gyre is negligible relative to the inflow and outflow of waters in distinct density classes on decadal timescales. To explore this connection, they compile an AMOC estimate at 45N based on an ensemble of reanalysis products referenced to satellite altimetry. The latitude 45N was chosen because it has good data coverage and is south of the outcropping of the AMOC’s lower limb. They find that the decadal variability of the AMOC at 45N is led by the surface-forced transformation north of this latitude by about 5 years. This is generally consistent with the results of Jackson *et al.* [18] and Le Bras *et al.* [73] discussed in §3 [18,73].

**Figure 6.**
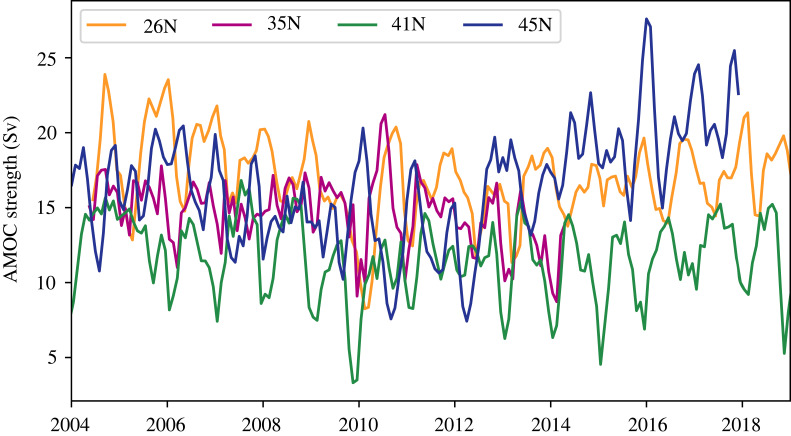
Monthly AMOC strength estimates from the RAPID program (26N, Moat *et al.* [77]), a mooring, altimeter and float synthesis at 35N (Le Bras *et al.* [73]), an altimeter and float synthesis at 41N [78], and a reanalysis and altimetry based dataset at 45N [74]. All AMOC time series shown are in depth coordinates.

In order to compare these studies, all AMOC time series are plotted on the same axis in figure 6. Note that all AMOC time series shown here are the maximum overturning in depth space as the 26N and 41N datasets are only available in this format (as opposed to in density space) [77,78]. All time series were averaged so that they have the same temporal resolution for this comparison. The RAPID 26N AMOC data are provided with 10-day resolution and the Desbruyères *et al.* 45N AMOC has monthly resolution. In order to match the resolution of the 35N and 41N datasets, the RAPID data were first box-averaged to monthly resolution and a 3-month rolling-average was applied to both the 26N and 41N datasets. Before about 2012, all time series have similar magnitudes and are declining, though there is significant seasonal variability in each time series. The 35N time series ends in 2014, when the Line W mooring program ended. The 45N time series begins increasing in 2012 and the 26N follows thereafter. As discussed by Desbruyères *et al.* [74] and Moat *et al.* [77], this is consistent with the southward propagation of high-latitude water mass transformation anomalies as dense LSW production increased again starting in 2014. By contrast, the 41N AMOC stays weaker than all other time series. The methodology used to construct the 41N and 45N datasets is similar in that both use thermal wind, are based largely on Argo float data and satellite altimetry. They differ in that the 41N dataset is referenced to Argo float trajectories, which are corrected for eddy-noise using altimetry, while the 45N dataset is referenced to altimetry-derived surface geostrophic velocities. The details of the disagreement in variability between the 41N and 45N datasets warrants further research.

Finally, we note that no direct link has been found between AMOC strength in the subpolar North Atlantic (as measured by OSNAP) and LSW formation [30]. This is in part due to the fact that LSW spreads throughout the subpolar region after its formation, including into the eastern subpolar North Atlantic [52]. Hence the geometry is fundamentally different than to the south, where the largest amount of LSW is always found on the western boundary. Furthermore, subpolar overturning is sensitive to the definition of the maximum of the overturning streamfunction as discussed in §2. Additionally, the net subpolar overturning strength value is not necessarily reflective of which water masses are found in the lower limb of the overturning circulation (figure 3). For example, subpolar overturning may be strong because of high subpolar mode water formation or overflow water export rather than LSW formation. As the climate warms and water mass formation at high-latitudes changes, it will be important to examine not only AMOC strength, but also its structure.

## 5. Summary and outlook

In this paper, I review and synthesize our current observational understanding of LSW export in boundary currents from the subpolar to the subtropical North Atlantic as well as potential connections to the AMOC. I first clarify the role of LSW in the subpolar overturning circulation in light of recent OSNAP results highlighting the importance of the eastern subpolar gyre (§2) [34,79]. The relative magnitude of the LSW contribution to the overturning is sensitive to how the overturning streamfunction is defined. An analysis focusing on the overturning circulation’s lower limb below the mean isopycnal of maximum overturning shows a convergence of about 5 Sv into the LSW density class in the Labrador Sea, which is consistent with previous studies [35,36]. I also find that transformations in the Labrador Sea add 2 Sv to the lower limb defined in this way.

LSW is exported in the Labrador Sea’s DWBC on an annual basis and decadal variability in LSW formation is reflected in these exported waters (§2) [23,37]. About half of the LSW that is exported is thought to be formed by deep convection in the interior of the Labrador Sea, while the other half is formed within the boundary current itself (figure 4) [39,43,45]. This suggests that there is an outsize role for boundary current convection that requires further study. LSW formed in boundary currents has been found to be exported rapidly within a few months of its formation [33,39], while LSW formed by deep convection persists in basin interiors from year-to-year and impacts interannual water mass transformation [80].

Moving southward, the DWBC is the most coherent pathway for LSW into the subtropics (§3) [12,13,50]. There is general consensus that it takes about 5 years for LSW density anomalies to be advected to 40–45N and about 10 years to the 26N RAPID array (figure 5) [61,74]. This result holds across many scientific methods, including water mass anomaly tracking, anthropogenic tracers and Lagrangian studies.

The connections between LSW formation and AMOC decadal variability are less clear (§4). There is no documented connection between LSW and subpolar overturning variability, partly because LSW spreads throughout the subpolar North Atlantic and partly because subpolar AMOC strength does not necessarily reflect the details of its complex overturning streamfunction structure, which includes ongoing (density-compensated) water mass transformations from the eastern subpolar to the Labrador Sea (figures 2 and 3) [30,35,81] (see §§2 and 4 for further discussion).

In the subtropics, Le Bras *et al.* [73] have recently found an AMOC decline at 35N associated with changing LSW properties and a DWBC decline from 2004 to 2014 [73]. This is consistent with AMOC declines and subsequent recoveries observed at 45N [74] and 26N [77]. However, this picture is muddled when all AMOC time series are plotted together (figure 6), and there is particular disagreement between AMOC time series at 41N and 45N [74,78], which requires further investigation. Note that only the 26N AMOC time series is based almost entirely on direct *in situ* observations.

Elucidating the connection between LSW formation and export and the AMOC is limited by the scarcity of AMOC observations. It is worth taking a step back and examining why this connection is of interest in the first place. If there were a clear connection, it would offer some predictability for long-term AMOC variability and hence climate variability. Potentially just as importantly for climate variability, LSW formation and export in the lower limb of the AMOC is a significant pathway for dissolved gases such as carbon dioxide and oxygen into the deep ocean [1,2]. Therefore, understanding the dynamics of how dissolved gasses enter the lower limb of the overturning circulation is of central importance and calls for direct observations of dissolved gasses alongside classical physical variables. Such efforts are underway through GOHSNAP in the subpolar North Atlantic and the Atlantic BiogeoChemical fluxes program at the RAPID array [51,82] and should become a standard part of AMOC observations and analyses. Given the significance of the DWBC pathway and the fact that it is undersampled by Biogeochemical Argo floats, moored dissolved gas measurements at targeted western boundary locations should be an AMOC observing priority.

## Data Availability

The Overturning in the North Atlantic Program observations shown in figures 2 and 3 can be accessed at www.o-snap.org/observations/data/. Readers are referred to the articles cited in the figure captions for the original data sources.
